# Meta-Analysis of Microbial Communities in Hot Springs: Recurrent Taxa and Complex Shaping Factors beyond pH and Temperature

**DOI:** 10.3390/microorganisms8060906

**Published:** 2020-06-16

**Authors:** Francisco L. Massello, Chia Sing Chan, Kok-Gan Chan, Kian Mau Goh, Edgardo Donati, María Sofía Urbieta

**Affiliations:** 1 CINDEFI (CCT, La Plata-CONICET, UNLP), Facultad de Ciencias Exactas, Universidad Nacional de La Plata, La Plata, 1900 Buenos Aires, Argentina; massello.f@gmail.com (F.L.M.); donati@quimica.unlp.edu.ar (E.D.); 2 Faculty of Biosciences and Medical Engineering, Universiti Teknologi Malaysia, Skudai 81310, Malaysia; chanchiasing@gmail.com (C.S.C.); gohkianmau@utm.my (K.M.G.); 3 Division of Genetics and Molecular Biology, Faculty of Science, Institute of Biological Sciences, University of Malaya, Kuala Lumpur 50603, Malaysia; kokgan@um.edu.my

**Keywords:** hot springs, extreme environments, microbial communities, extremophiles, amplicon sequencing, Caviahue-Copahue, Domuyo

## Abstract

The study of microbial communities from extreme environments is a fascinating topic. With every study, biologists and ecologists reveal interesting facts and questions that dispel the old belief that these are inhospitable environments. In this work, we assess the microbial diversity of three hot springs from Neuquén, Argentina, using high-throughput amplicon sequencing. We predicted a distinct metabolic profile in the acidic and the circumneutral samples, with the first ones being dominated by chemolithotrophs and the second ones by chemoheterotrophs. Then, we collected data of the microbial communities of hot springs around the world in an effort to comprehend the roles of pH and temperature as shaping factors. Interestingly, there was a covariation between both parameters and the phylogenetic distance between communities; however, neither of them could explain much of the microbial profile in an ordination model. Moreover, there was no correlation between alpha diversity and these parameters. Therefore, the microbial communities’ profile seemed to have complex shaping factors beyond pH and temperature. Lastly, we looked for taxa associated with different environmental conditions. Several such taxa were found. For example, *Hydrogenobaculum* was frequently present in acidic springs, as was the Sulfolobaceae family; on the other hand, *Candidatus* Hydrothermae phylum was strongly associated with circumneutral conditions. Interestingly, some singularities related to sites featuring certain taxa were also observed.

## 1. Introduction

Geothermal areas have proven to be interesting environments for diverse scientific disciplines [1,2,3]. Many researchers have focused on the relation between microbial communities and environmental factors while taking different approaches, yet the general belief is that temperature is the main factor that drives the community structure [4,5,6]. It has also been reported that extreme conditions decrease the biodiversity [7,8]. However, it may not be that simple, as Power et al. [9] and Uribe-Lorio et al. [10] have shown that pH has a strong influence in the communities’ structure, since the samples in both studies could be split in two clusters: one with acidic pH and the other one with circumneutral conditions. Power et al. [9] also showed that temperature influence was significative only at values above 70 °C, which suggests that other explicatory variables may arise if the range considered for pH and temperature is narrow. For instance, Mathur et al. [11] compared acidic and thermophilic (pH 2 and 75 °C) springs from Yellowstone National Park (USA) and found that 95% of the variance in the biodiversity could be assigned to sediment composition, with only a small portion of the variance being assigned to temperature. Similarly, Purcell et al. [12] studied hyperthermophilic and alkaline (pH 7.5–9 and 75–90 °C) hot springs of Thailand and concluded that the combined conditions of temperature and sulfide concentration were the most important factors that drove the diversity of bacteria and archaea. On the other hand, some authors advocate for the idea of a biogeographical distribution of microorganisms and have shown evidence of correlation between species diversity and geographic distance [13,14]. In summary, the relation between microbial communities and environmental factors is undoubtedly complex and yet to be dilucidated.

Interestingly, and overcoming the great variability that exists between hot springs, several microorganisms have been identified that appear to be ubiquitous and typical of these habitats. Thomas Brock [15] made an extraordinary survey of thermophilic habitats around the world and described the most frequent microorganisms found. He particularly mentioned the cyanobacteria *Synechococcus*, the photosynthetic bacteria *Chloroflexus*, chemolithotrophs related to sulfur of the genera *Acidithiobacillus* and *Sulfolobus*, the heterotrophic bacteria *Thermus* and the archaea *Thermoplasmata*, among others. Since then, with taxonomic reclassifications in between, these microorganisms were found the majority of times in different hot springs. A major addition to this group of thermophilic prokaryotes was the phylum Aquificae. The phylum Aquificae was first reported in 1992 [16] and classified in 2001 [17]; since then, it has been found in many terrestrial and marine geothermal systems [18]. Members of this phylum are obligate or facultative autotrophs with great chemolithotrophic metabolism variants, from the use of H_2_, S_0_, S^2−^, S_2_O_3_^2−^, SO_3_^2−^, Fe^2+^ or AsO_3_^3−^ as electron donor to O_2_, NO_3_^−^, SO_3_^2−^, Fe^3+^, AsO_4_^3−^ or SeO_3_^2−^ as electron acceptors. For example, *Aquifex* is capable of hydrogen oxidation and, using oxygen at very low concentration as an electron acceptor, it produces water at temperatures up to 95 °C [16]. Extraordinary abilities are not exclusive to Aquificae but are characteristic of all extremophiles; for example, the potential of the abovementioned *Thermus* genus and the Taq Polymerase used in PCR reactions is widely known. The biotechnological interest in thermostable enzymes is well reported [19,20], but extremophiles are also of ecological, evolutional and astrobiological interest because of their relationship with ancestral lineages of life on Earth [21,22,23,24].

Caviahue-Copahue and Domuyo are two volcanic-geothermal areas with distinct environmental conditions, located in the Cordillera de los Andes in Neuquén, Argentina. Caviahue-Copahue is mainly acidic and has high sulfur and iron content. The area is subjected to the activity of the Copahue volcano, which has shown several eruptive cycles in recent years (2000, 2012–2013 and 2018), resulting in an extremely unsteady environment. Hence, geothermal ponds of the region are in constant change in terms of physical appearance, size, pH, temperature and ionic content; moreover, we have detected the disappearance and the outcrop of springs from year to year. On the other hand, the Domuyo geothermal area presents circumneutral conditions and high temperatures. Unlike Copahue, Domuyo is considered an inactive volcano whose geothermal activity is fault-controlled [25]; it presents magmatic chambers near the base of the hill and several surface manifestations as geysers, fumaroles and hot springs. Our research group has been studying the prokaryotic diversity of Caviahue-Copahue for more than twenty years through different approaches, from classical culture-based microbiology [26,27] to culture-independent techniques such as cloning and high-throughput amplicon sequencing [28,29,30,31]. We know that the microbial community is dominated by iron- and sulfur-related microorganisms such as *Acidithiobacillus*, *Sulfobacillus*, *Sulfolobus* and *Acidianus*. Remarkably, we have isolated and characterized two novel autochthonous microbial species, the thermoacidophilic archaea *Acidianus copahuensis* [32] and the sulfate-reducing bacteria *Desulfotomaculum copahuensis* [33]. Conversely, Domuyo has been scarcely studied, and its prokaryotic diversity has not been surveyed yet; there is only one report regarding hydrolytic enzymes in thermophilic bacteria [34].

In this work, we surveyed the microbial diversity of three hot springs with distinct conditions of pH and temperature from Caviahue-Copahue and the until-now unexplored Domuyo geothermal area. Besides, we collected data from 94 other hot springs around the world and analyzed the influence of pH and temperature on the microbial diversity and structure. Finally, we looked for frequent microbial taxa associated with distinct environmental conditions. As far as we know, this is the first meta-analysis that explores high-throughput sequencing data of microbial communities of hot springs from several studies and from such distinct locations.

## 2. Materials and Methods 

### 2.1. Sample Collection and Physicochemical Determination

**Figure 1:**
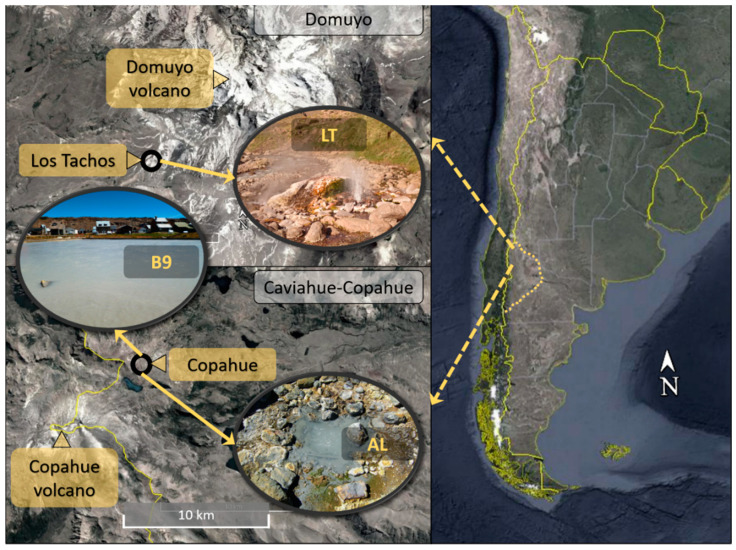
Location of sampling sites in Neuquén, Argentina.

Two volcanic regions in Neuquén, Argentina, were studied (Figure 1). The Caviahue-Copahue region consists of several acidic rivers and geothermal manifestations and is governed by the activity of Copahue volcano; on the other hand, Domuyo is also a volcanic region, but the site presents circumneutral conditions. Two hot springs were sampled from Caviahue-Copahue, namely Agua de Limón (AL) (36.691° S, 70.546° W) and Baño 9 (B9) (37.816° S, 71.096° W), while one sample was taken from a geothermal pond at Los Tachos (LT) (36.691° S, 70.546° W), Domuyo. Water and sediment samples were taken with sterile plastic jars and preserved at −20 °C.

Physicochemical parameters were measured in situ. Temperature, electrical conductivity, redox potential, dissolved oxygen and pH were measured using a Hanna HI 8424 (Hanna, Smithfield, RI, USA) portable instrument accurately calibrated against calibration standards. Arsenic concentration was measured with the Merckoquant Arsenic Test colorimetric method commercial kit with test strips (Merk, Darmstadt Germany). Sulfide concentration was measured with a HACH DR-850 colorimeter (HACH, Loveland, CO, USA) using the methylene blue method. Later, metal content (Na, K, Ca, Cd, Zn, Fe, Cu, Ni, Pb, Co and Cr) was measured by atomic absorption spectrophotometry using a Shimadzu AA-6650 spectrophotometer (Shimadzu, Kioto, Japan).

### 2.2. DNA Extraction and Amplicon Sequencing

Total DNA from the three samples was extracted using PowerSoil DNA Isolation Kit (MOBIO Laboratories Inc., Carlsbad, CA, USA) according to the manufacturer’s instructions. The DNA quality was estimated using Nanodrop (Thermo Scientific, Waltham, MA, USA).

PCR amplification of the hypervariable regions V3–V4 of the 16S rRNA gene was performed using pair of primers, namely S-D-Bact-0341-b-S1 and S-D-Bact-0785-a-A-21 (CCTACGGGNGGCWGCAG and GACTACHVGGGTATCTAATCC, respectively) containing Illumina overhang adapter sequences. This is a suitable pair of primers for the amplification of the 16S rRNA gene of bacteria and archaea [35]. Amplicons were sequenced on Illumina MiSeq sequencer platform as 300 bp paired-end reads at the High Impact Research Institute at the University of Malaya, Malaysia. The sequencing resulted in 242,246, 383,332 and 296,146 raw paired-end reads for samples AL, B9 and LT, respectively. The sequences were deposited in NCBI under the accession numbers SRR9035351, SRR9035350 and SRR9035352.

### 2.3. Sequences Processing

The raw sequencing data were processed in R [36], using the DADA2 package [37] to infer amplicon sequence variants (ASVs). The pipeline was followed using the standard parameters proposed by Callahan et al. [37]. Reverse read data were of poor quality, hence, only forward reads were used. At the end of the process, and after removing chimera sequences, 115,712; 214,773 and 155,053 clean amplicon sequences were obtained for AL, B9 and LT, respectively. In each case, they represented about 50% of the input data and more than 95% of the filtered sequences. These clean sequences were used for the inference process of ASVs, which resulted in 822 sequences.

Taxonomic assignment was performed using the Silva nonredundant training set (v. 132), and species were assigned based on exact match between the ASVs and the reference strains in the Silva database. ASVs with unclassified kingdom and those assigned as Eukaryote were removed. At the end, we obtained 811 ASVs.

Rarefaction curves were constructed using vegan package in R [38], and two alpha diversity indexes were calculated, namely Shannon and inverse Simpson, using the phyloseq package [39].

### 2.4. Hot Spring Data Collection

We explored the NCBI Sequence Read Archive (SRA) for high-throughput sequencing data of hot spring environments. We collected the raw sequencing data of 94 hot spring sites from seven studies [9,40,41,42,43,44,45]. Sites were named and numbered according to their location, which included Malaysia (MS); New Zealand (NZ); Raoul Island, New Zealand (RI); Sikkim, India (SK); Tengchong, China (TC); Uzon Caldera, Russia (UZ); and Yellowstone National Park, USA (YS). Sites were later classified according to their pH and temperature. Factor levels for pH were ’A’ for acidic samples (pH < 5) and ’N’ for circumneutral samples (pH > 5); temperature categories were ’T’ for samples with temperature lower than 70 °C and ’H’ for hyperthermophilic samples (temperature > 70 °C). Supplementary Table S1 shows full collection metadata.

We processed the data as in Section 2.3 and inferred the ASVs of each sample. Later, as data came from different sequencing processes and targeted different regions of the 16S rRNA gene, we proceed with a phylotype approach. To do this, we collapsed and aggregated the ASVs table at the genus level. Including the three samples of this study, we obtained 1850 phylotypes from the 97 hot springs.

### 2.5. Hot Springs Analysis

Alpha diversity indexes were calculated for each sample based on their ASVs table (not on the phylotypes). Spearman’s rank correlation coefficient was calculated using the cor.test function in R to test for a correlation between alpha diversity, pH and temperature. Bray–Curtis dissimilarity coefficients were calculated based on the relative abundances. A Mantel test was performed with 9999 permutations to test if this dissimilarity matrix co-varied with the pH and temperature. For this, temperature and pH from the sites were standardized and a Euclidean distance-based matrix was calculated for each. Mantel statistics were based on Pearson’s product-moment correlation using the mantel function in vegan R package.

A nonmetrical dimensional scaling plot (NMDS) was produced. The dissimilarity matrix was also used to test different explicatory models. Firstly, we estimated the dispersion of the classified variables (pH and temperature) using the betadisper function in R; then, we evaluated an ordination model through a permutational analysis of variance (PERMANOVA) with the adonis function in R.

The most abundant taxa at different taxonomic ranks were represented in a heatmap to analyze the occurrence in the groups. 

Indicator value index for each taxon to each group was calculated as described by Dufrene and Legendre [46] using the indicspecies package in R [47]. The test of significance was performed with 5000 permutations.

## 3. Results

### 3.1. Site Description and Physicochemical Characteristics of the Samples

Three hot spring ponds from two volcanic regions in Neuquén, Argentina, were sampled (Figure 1). Sites were chosen based on different conditions of pH and temperature. Agua de Limón (AL) is a hot spring site in Caviahue-Copahue, and the pond sampled presented acidic (pH = 2.4) and hyperthermophilic conditions (71 °C); Baño 9 (B9), also located in Caviahue-Copahue, had acidic pH (2.3) but lower temperatures than AL (57 °C); Los Tachos (LT), in Domuyo, is a site with circumneutral conditions, and the pond sampled presented pH of 7.6 and hyperthermophilic temperatures (75 °C).

Table 1 summarizes the physicochemical parameters measured. Both samples from Caviahue-Copahue, AL and B9, presented negative Eh values, low dissolved oxygen (0.3 mg/L) and high iron content (AL, 76.13 mg/L; B9, 17.59 mg/L). Other metals detected were Cu (0.66 mg/L), Mg (0.76 mg/L), Mn (0.54 mg/L), Pb (0.34 mg/L), Na (24.14 mg/L) and K (16.14 mg/L) in AL; while the B9 sample presented Ca (1.45 mg/L), Mg (3.02 mg/L), Mn (2.42 mg/L), Pb (0.29 mg/L), Na (38.27 mg/L) and K (6.72 mg/L). On the other hand, the sample from Domuyo, LT, presented higher dissolved oxygen (8.2 mg/L) and a positive Eh value. Regarding metals, iron content was below the equipment’s sensitivity (0.2 mg/L), while Ca (28.40 mg/L), Mg (2.48 mg/L), Mn (5.61 mg/L), Na (1418.52 mg/L) and K (109.91 mg/L) were detected.

### 3.2. Sequencing Analysis and Microbial Diversity

**Figure 2:**
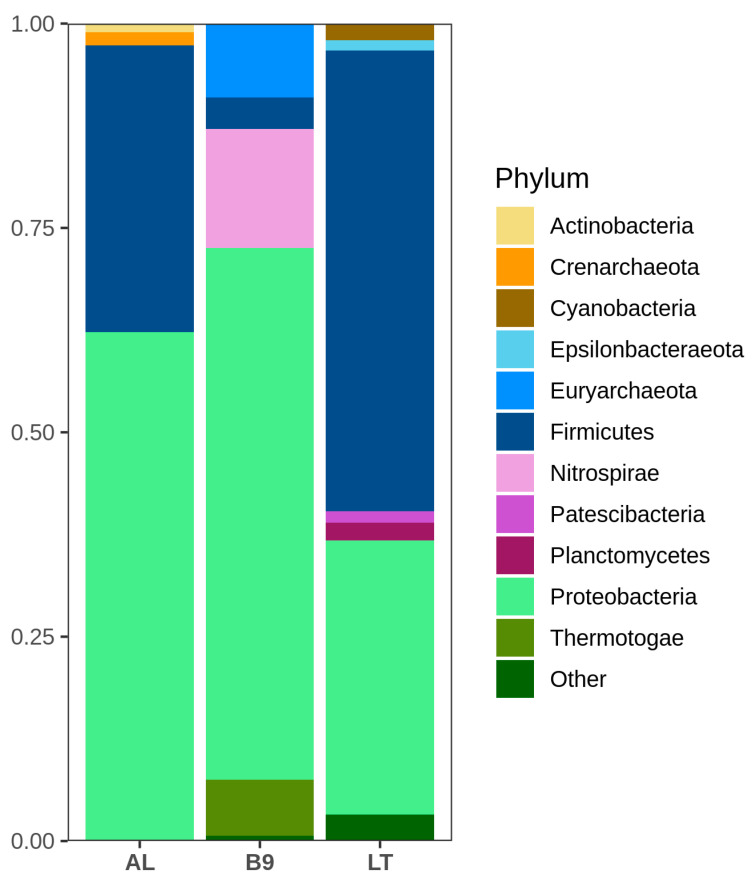
Relative abundance of phyla in the microbial communities of Agua de Limón (AL), Baño 9 (B9) and Los Tachos (LT). Phyla with abundance lower than 1% were grouped as “Other”.

A total of 921,724 raw sequences were obtained and processed following the DADA2 pipeline, resulting in 485,538 clean sequences which were taxonomically classified in 811 ASVs. At kingdom level, B9 presented the highest proportion of archaea (9.2%), while archaea represented 1.7% in AL. On the other hand, the LT microbial community was composed of only bacteria. At phylum level, Proteobacteria and Firmicutes were the most represented taxa in all three samples. Proteobacteria was the dominating phylum in Caviahue-Copahue communities, representing 65% and 62% of B9 and AL, respectively, while Firmicutes represented just 3% of B9 and 35% of AL. Conversely, LT was dominated by Firmicutes (56%), with Proteobacteria being the second most abundant phylum (33%) (Figure 2).

For the B9 community, the most abundant ASVs were classified as *Acidithiobacillus* sp. (ASV 1: 57%), *Leptospirillum* sp. (ASV 5: 14%), uncultured Thermoplasmatales (group BSLdp215) (ASV 6: 8%), *Mesoaciditoga* sp. (ASV 8: 6%), *Acidithiobacillus thiooxidans* (ASV 9: 3%), *Sulfobacillus thermotolerans* (ASV 3: 3%) and *Acidithiobacillus* spp. (ASV 13: 2%; ASV 17: 1%). AL’s most abundant taxon was also classified as *Acidithiobacillus*, but it was a different ASV than the dominant one in B9 (ASV 2: 57%). In AL, the second and the third most representative taxa were shared with B9, namely *Sulfobacillus thermotolerans* (ASV 3: 34%) and *Acidithiobacillus* sp. (ASV 13: 2%). Another *Acidithiobacillus* sp. (ASV 23: 1%) and an *Acidimicrobium* sp. (ASV 30: 1%) completed this community. It is worth noting that just two taxa, *Acidithiobacillus* sp. and *Sulfobacillus thermotolerans*, represented more than 90% of this community. On the other hand, the LT community was much richer. Here, the most abundant ASV belonged to the anaerobic family *Lachnospiraceae* (ASV 4: 28%), followed by *Sphingomonas kwangyangensis* (ASV 7: 10%), Clostridium_sensu_stricto_13 (ASV 10: 5%), *Pannonibacter* sp. (ASV 11: 5%), *Fusibacter* sp. (ASV 12: 4%), *Desulfovibrio* sp. (ASV 14: 4%), *Herbinix* sp. (ASV 15: 3%) and several other taxa that presented abundances above 1%.

We constructed a rarefaction curve for each sample (Supplementary Figure S1) and calculated two alpha diversity indexes: Shannon and inverse Simpson. The rarefaction curves reached a plateau in all three cases; therefore, samples were well represented. Both analyses, the curve and the indexes, reveal that the LT community presented much more alpha diversity (richness and evenness) than the two Caviahue-Copahue communities.

### 3.3. Hot Spring Microbial Structure and Shaping Factors

A collection of hot springs microbial communities was created from amplicon sequencing data submitted in the NCBI Sequence Read Archive (SRA) to study the roles of pH and temperature as shaping factors. The metadata of this collection can be found in Supplementary Table S1. A Mantel test corroborated the hypothesized covariance of the community profile with pH (*R* = 0.36, *p* = 0.0001) and temperature (*R* = 0.17, *p* = 0.0001). Therefore, the samples could be classified according to these conditions.

**Figure 3:**
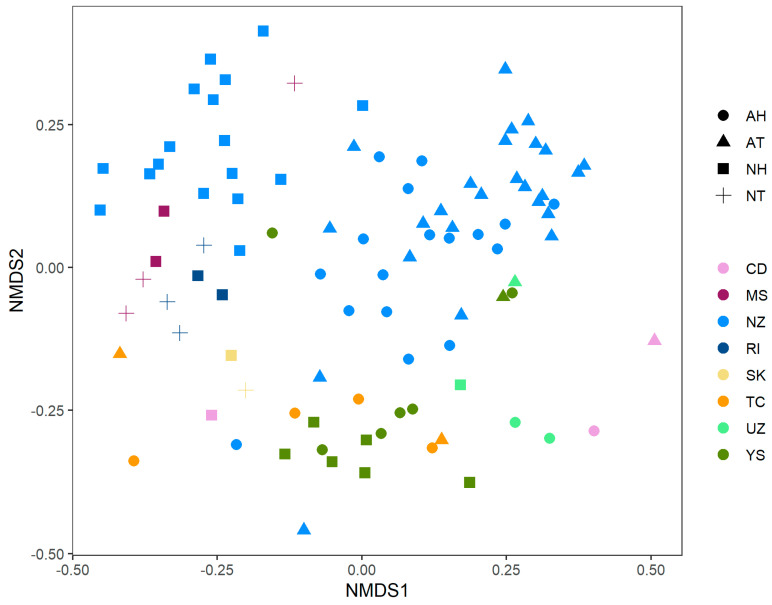
Nonmetrical dimensional scaling plot NMDS of the collection of hot spring microbial communities. Colors correspond to data sources (see Supplementary Table S1), while shapes map for pH and temperature groups. Factor levels for pH were ‘A’ for acidic samples (pH < 5) and ’N’ for circumneutral samples (pH > 5. Temperature categories were ‘T’ for samples with temperature lower than 70 °C and ’H’ for hyperthermophilic samples (temperature > 70 °C).

To explore how the phylogenetic structures of these hot springs relate with each other we performed an NMDS (Figure 3). As indicated by the Mantel test, the plot showed an influence of pH and temperature, with higher impact of pH; however, there was a clear tendency for samples from the same study to cluster together despite their physicochemical conditions.

The previous observations were firstly tested with a dispersion test. We found that the intradispersion between samples grouped by the combined conditions of pH and temperature was significant (alpha = 0.01); therefore, the distance between these groups could not be tested. Similarly, grouping only by temperature showed a significant dispersion; hence, this variable was discarded as well. On the other hand, pH category and the study source of the data (namely “Source”, see Supplementary Table S1) could be explicatory variables.

With this in mind, we performed a PERMANOVA to test an ordination model based on pH and data source. Both variables were significative (alpha = 0.001), but the model explained only 27% of the microbial profile (19% explained by the source and 8% explained by pH).

**Figure 4:**
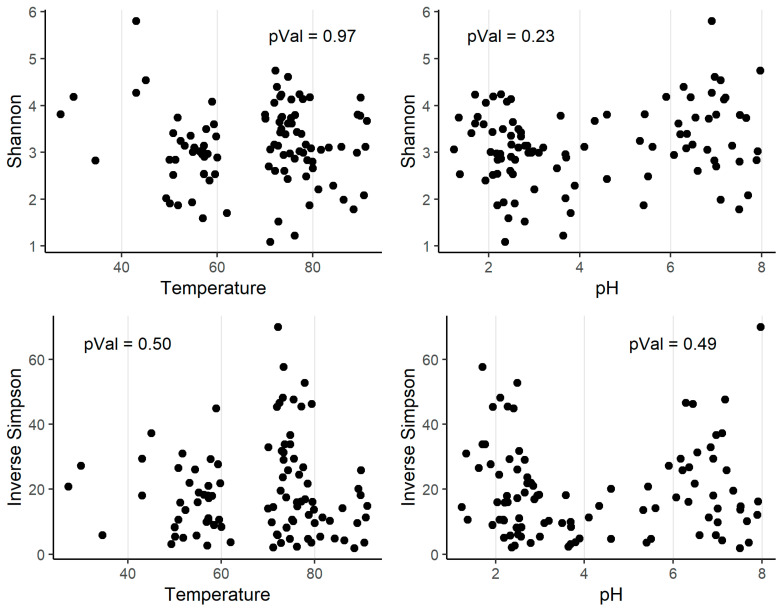
Alpha diversity indexes and their correlations with pH and temperature (°C). The *p*-value of the Spearman’s rank correlation coefficient (Rho) is shown.

The alpha diversity indexes (Shannon and inverse Simpson) were calculated for all the communities in the collection (Supplementary Table S2). As can be seen, the circumneutral sample (LT) had much more alpha diversity than the acidic samples (AL and B9). Then, we computed the Spearman’s rank correlation coefficient to test if there was a relation between alpha diversity and pH or temperature. We found that there was no significant correlation between any of these parameters (alpha = 0.01) (Figure 4).

### 3.4. Co-Occurrence of Taxa Across Samples

**Figure 5:**
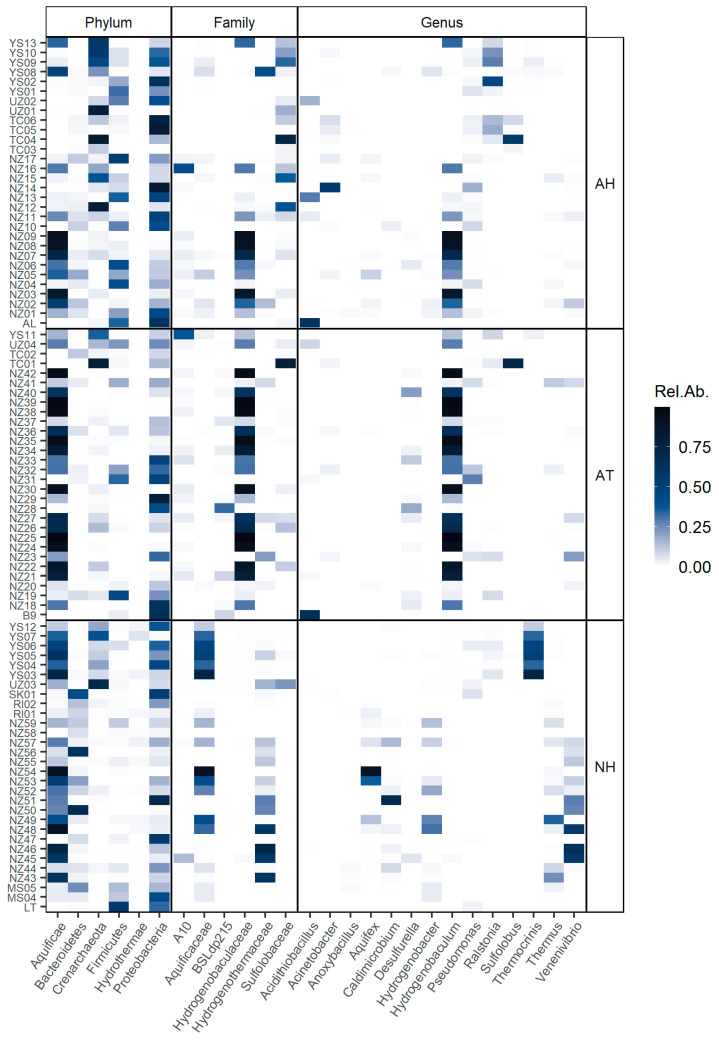
Heatmap of the relative abundance of taxa at different ranks across samples and conditions. Taxa are named and ranked (x-axis) are according to the Silva database. Sample names (y-axis) are fully described in Supplementary Table S1. The first two letters of each sample name correspond to the location of the sample: Malaysia (MS); New Zealand (NZ); Raoul Island, New Zealand (RI); Sikkim, India (SK); Tengchong, China (TC); Uzon Caldera, Russia (UZ); and Yellowstone National Park, USA (YS). LT, B9 and AL are the samples from Caviahue-Copahue and Domuyo described in this study. Sample conditions are described as acidic and hyperthermophilic (AH), acidic and thermophilic (AT) or circumneutral and thermophilic (NH).

We analyzed the occurrence of the taxa across the different samples and found that there were some frequent taxa in certain conditions (Figure 5). The *Hydrogenobaculum* genus appeared to be highly abundant in acidic conditions, while Thermoplasmatales group A10 was also present in most acidic samples but in less abundance. *Acidithiobacillus* was highly abundant in some of the acidic samples, particularly in the hot springs from Caviahue-Copahue. The *Sulfolobaceae* family was abundant in acidic and hyperthermophilic samples; also abundant but less representative was the genus *Ralstonia*, while *Pseudomonas* was present in almost every acidic and hyperthermophilic sample but in low abundance. *Desulfurella* and the Thermoplasmatales group BSLdp215 were the most common taxa in acidic and thermophilic conditions. In circumneutral pH, the diversity of taxa was higher, but some genera were frequent across these samples, as was the case of *Thermus*, *Hydrogenobacter* and *Caldimicrobium*. *Thermocrinis* was highly abundant, but only in samples from Yellowstone National Park, while *Venenivibrio* was mostly representative of New Zealand neutral hot springs.

The indicator value (IndVal) for each taxon was calculated according to Dufrene and Legendre [46] (Table 2). IndVal is the product of two components, A (specificity) and B (fidelity). Component A nears 1 when the sample is present only in a given group, while component B nears 1 when the sample is present in all the sites of a given group. Here, we report the square root of IndVal (Stat). As had been observed in the heatmap (Figure 5), *Hydrogenobaculum* was strongly associated with acidic samples (Stat = 0.87), being highly specific for this group (A = 0.99). *Sulfolobaceae* family (Stat = 0.72) and, to a lesser extent, *Acinetobacter* (Stat = 0.64) were associated with acidic and hyperthermophilic temperatures (group AH). On the other hand, *Desulfurella* (Stat = 0.72) and the uncultured Thermoplasmatales group BSLdp215 (Stat = 0.68) were indicators of acidic and thermophilic springs (group AT). Several taxa were significantly associated with circumneutral samples (group N), especially the Hydrothermae phylum (Stat = 0.83) and the genera *Thermus* (Stat = 0.76), *Caldimicrobium* (Stat = 0.73) and *Hydrogenobacter* (Stat = 0.70). *Anoxybacillus* was the only taxa associated with extremely high temperatures (group H), appearing only in samples with these temperature conditions regardless of pH (A = 1.00); however, since it was not recurrent in all the hyperthermophilic samples (B = 0.32), its indicator value was low (Stat = 0.57), which suggests that this genus was not a good indicator of hyperthermophilic conditions.

## 4. Discussion

Hot springs are interesting environments due to their extreme conditions and the versatile microorganisms that inhabit them. In this study, we surveyed the microbial community of three hot springs from two volcanic areas in Neuquén, Argentina. The Caviahue-Copahue region has been extensively studied by this research group [28,29,30,31,48,49] and many others [50,51,52,53,54]; on the other hand, Domuyo has been scarcely visited and there is only one report related to hydrolytic enzymes in thermophilic bacteria isolated from this site [34]. The hot springs sampled were chosen due to their differential conditions of pH and temperature: AL and B9 were acidic ponds, but with different temperatures (71 °C and 57 °C, respectively), while LT was a circumneutral pond with high temperatures (75 °C). All three hot springs had relatively low concentration of metals; however, we highlighted the abundance of iron and lead in Caviahue-Copahue. As these are both geothermal environments, it must be considered that the conditions may vary significantly, subject to the geothermal activity.

To survey these three communities using high-throughput amplicon sequencing, we choose the ASVs approach recently developed by Callahan et al. [37] in contrast to the classical OTU-based analysis. OTU-based analysis has some major drawbacks: the difficulties in reproducing and comparing results, the dependence on a database and the overestimation of taxa [55]. On the other hand, ASVs analysis considers the quality and the error rate of sequences in a sample resulting in a method that is reproducible and consistent.

We analyzed the taxonomic classification of the ASVs to predict the metabolic profile of the samples. The acidic communities of Caviahue-Copahue showed a preponderance of chemolithoautotrophic microorganisms associated to iron and sulfur, represented mostly by *Acidithiobacillus* spp. as has been largely reported in our previous studies. *Sulfobacillus thermotolerans*, a Gram-positive, aerobic bacteria capable of oxidation of ferrous iron, sulfur, tetrathionate and sulfur minerals [56], was found in both samples. In B9, there was a significant abundance of a *Leptospirillum* sp.; these bacteria are characterized by the ability to oxidize iron but not sulfur compounds [57]. In this community, we also found a taxon classified as *Mesoaciditoga* sp.; this genus has only one species reported, *Mesoaciditoga lauensis*, which has been described as a moderately thermoacidophilic heterotrophic bacterium with a pH range of 4.1–6.0 being suitable for growth [58]. Since the conditions in this hot spring were very acidic and there was likely low organic matter content, moreover, since the fragment sequenced did not match with the sequence reported for *M. lauensis* (we used the BLAST algorithm to test it), we believe that it may be another related species not yet isolated. Beyond that, all the taxa found agree with our previous studies of the Caviahue-Copahue area.

The LT community had more alpha diversity, as both Shannon and inverse Simpson indexes were higher than the acidic samples, revealing more richness and more evenness, respectively. There was an absolute dominance of chemoheterotrophic microorganisms, including both aerobic and anaerobic mesophilic and moderately thermophilic bacteria. The most abundant ASV, though just representing 28% of the community, was classified as *Lachnospiraceae*, an anaerobic chemoorganotrophic family which is phylogenetically heterogeneous [59]. Hence, the lack of resolution prevents us from doing any further discussion. The second most abundant taxon was *Sphingomonas kwangyangensis*, this species has twice been reported as isolated from cooling water systems in unpublished works (sequences JN377661.1 and EF693741.1). *Sphingomonas* genus comprises Gram-negative, chemoheterotrophic, strictly aerobic bacteria that are free-living in natural and man-made environments, including polluted and unpolluted sites [60]. Two additional genera were also identified in this sample, *Fusibacter* and *Herbinix*; the first one has been isolated from oiled environments and has arsenate-reducing potential [61,62], while the second one comprises thermophilic cellulose-degrading bacteria [63,64]. Conclusively, this first approach to the indigenous microbial community of Domuyo revealed a rich and interesting environment which could host new and biotechnologically useful organisms.

To analyze the relevance of pH and temperature as driving factors of the microbial community, and also to search for frequent taxa in these environments, we collected amplicon sequence data from other hot springs around the world. Most of the studies present conflicting reports as to whether pH or temperature is the major shaping factor [4,5,6,9,10]. On the other hand, some authors have proposed other significant variables. For instance, Mathur et al. [11] showed that sediment composition was the most explicative variable in the biodiversity of hot springs from the Yellowstone National Park, and Purcell et al. [12] concluded that sulfide concentration exerted a significative selection pressure on hot spring communities in Northern Thailand. In our work, we first tested our starting hypothesis that pH and temperature influence the microbial community. We did a Mantel test and found that, indeed, the phylogenetic distance between hot springs co-varied with both pH and temperature. Therefore, we classified the samples as acidic/circumneutral and thermophilic/hyperthermophilic. However, when we tested this categorization, we found that the dispersion between samples of the same temperature group was significative, and hence we concluded that a classification on those two levels (thermophilic and hyperthermophilic) was not representative enough and that the nature of temperature’s influence was more complex. On the other hand, samples could be grouped by pH as acidic and circumneutral. Surprisingly, exploring the NMDS plot, we observed that samples tend to cluster according to the source of the data, regardless of the physicochemical condition. Furthermore, when we tested the ordination model considering data source and pH, we found that the first variable was the most explicative. This result may imply that microbial communities have certain profiles characteristic of the location (which would include several variables, such as seasonal changes, UV exposure and soil characteristics), as Jones et al. [14] and Liu et al. [65] have reported, but it could also represent, a methodological bias in areas such as the sequencing technology, the amplified region of the 16S rRNA gene and the DNA and sample extraction and conservation protocols. Notably, the model tested could not explain much of the ordination (only 27%), suggesting that the communities’ profile must be shaped in a more complex way.

Remarkably, we found that there was no correlation between pH, temperature and alpha diversity. This contradicts the general belief and the reported relation [7,8]; however, currently, there is a discussion about the meaning and indicative value of alpha diversity indexes [66]. Regarding temperature, the range included in this collection was arguably narrow, since the samples were mostly thermophilic; however, pH range comprised well acidic and circumneutral values, therefore, the reported no correlation between alpha diversity and pH must be noted. Further studies of hot springs around the world with similar experimental design, more explicatory variables and wider ranges would be interesting to test and explore this observation.

Even though the communities’ profile showed a complexity of driving factors, there still could be some microorganisms (taxa) strongly associated to certain conditions of pH and temperature. These taxa would be interesting to study, since their ubiquity implies serious adaptation mechanisms that might have ecological and biotechnological potential. Most of the studies that deal with the distribution of microorganisms in hot springs rely only on isolation reports [15,67,68], with few exceptions [8,69]. This approach has the inevitable bias toward cultivable organisms and a selection filter due to growth medium composition. As far as we know, there is no meta-analysis that crosses data from different amplicon sequencing studies looking for recurring taxa in similar conditions of geothermal systems around the world.

We analyzed the occurrence of the taxa across samples and conditions in our collection. Genus *Hydrogenobaculum* showed to be frequently and significatively associated with acidic conditions, being present only in acidic samples. The order Aquificales (parent of *Hydrogenobaculum*) is widely spread in geothermal systems [18,70,71] and is considered an ancestral bacteria lineage [72]; some of these microorganisms are characterized by their ability to oxidize hydrogen to water. Surprisingly, neither this order nor the phylum Aquificae was present in Caviahue-Copahue samples, even though Aquificales was the most abundant taxon in hot springs from Baño 9 in previous studies using cloning techniques [29]. The *Sulfolobaceae* family was also associated with hyperthermophilic and acidic conditions, this family comprehends thermoacidophilic archaea with optimum growth conditions of temperature above 65 °C and pH lower than 3.5. These microorganisms have been isolated from extreme environments such as sulfur-rich volcanic and geothermal areas [73,74]. The *Desulfurella* genus was found to be frequent in hot springs with acidic pH and temperatures below 70 °C. The genus is described as anaerobic, chemolithotrophic, acidotolerant and moderately thermophilic, capable of reducing sulfur. *Desulfurella* spp. have been isolated from acidic environments, such as Tinto River in Spain [75] and Los Azufres geothermal field in Mexico [76], and from circumneutral pools at Uzon Caldera, Kamchatka [77]. Interestingly, BSLdp215, a group of uncultured Thermoplasmatales, was also associated with acidic and hyperthermophilic springs.

Circumneutral hot springs showed the usual occurrence of genera *Thermus*, *Caldimicrobium* and *Hydrogenobacter*. The last one belongs to the aforementioned Aquificae phylum, typical of these environments. *Thermus* spp. have been found in several thermal habitats, both natural and artificial. These bacteria are chemoorganotrophs and have optimum pH of about 7.0–8.5 and optimum temperature of 65–75 °C [78]. *Thermus* spp. have been extensively studied and are of biotechnological interest due to the stability of their enzymes and their rapid growth yield [79,80,81]. *Caldimicrobium* is an extremely thermophilic genus (optimum temperature around 75 °C) found usually in springs from the volcanic region of Uzon Caldera, Kamchatka [82]. The genus consists of only two species, both chemolithoautotrophic sulfur-oxidizing bacteria [83,84]. Lastly, the taxon OPB56 was also associated with circumneutral springs. This uncultured clade is believed to be part of the Chlorobi phylum and has been found in hot springs in Yellowstone National Park and Japan, yet its classification is still being studied [85,86]. 

Interestingly, the taxon most associated with circumneutral and hyperthermophilic conditions was the Hydrothermae phylum. This is a Candidatus phylum (yet to be cultured) proposed by Jungbluth et al. [87] after sequencing the metagenomes of samples from the subseafloor on the Juan de Fuca Ridge. Most of the amplicon sequences assigned to this phylum in the Silva database belong to geothermal sites around the world. Not much is known about these bacteria, yet they seem to be ubiquitous in these habitats.

Finally, four singularities must be highlighted. First, *Acidithiobacillus*, a genus of iron/sulfur-oxidizing bacteria found in several acidic environments worldwide and mostly related to mine activity [88], dominates Caviahue-Copahue acidic springs, as has been reported by this research group through the years; however, only the acidic springs of Uzon Caldera showed a significant presence of the genus as well. In Yellowstone National Park samples, *Acidithiobacillus* was found, but in low abundance, which agrees with the rare identification of the genus in this environment (this can be seen in the geographical occurrence of *Acidithiobacillus* as analyzed by Nuñez et al. [89]). Surprisingly, no members of *Acidithiobacillus* were found in the acidic springs of New Zealand, although there was a high abundance of Gammaproteobacteria, which leads to the possibility of a resolution bias due to different sequencing approaches. Second, the circumneutral springs of Yellowstone National Park feature a unique abundance of *Thermocrinis*, an Aquificales hyperthermophilic genus isolated for the first time in that same geothermal area [90]. Third, in New Zealand’s circumneutral hot springs, there was a special abundance of *Venenivibrio*, a thermophilic hydrogen-oxidizing genus in the Aquificales order which was also isolated in that same country at Champagne Pool, Waiotapu [91]. Lastly, *Pseudomonas* was found in most acidic and hyperthermophilic sample in the collection. While this genus is considered ubiquitous, a *Pseudomonas* species capable of growing at temperatures above 70 °C and low pH has not been described or isolated. This result suggests that a deeper characterization of the known species is needed or that there are *Pseudomonas* species yet to be discovered in thermoacidophilic environments.

## 5. Conclusions

We assessed the microbial diversity of three hot springs from Neuquén, Argentina. In the acidic samples, chemolithotrophic microorganisms prevailed, such as *Acidithiobacillus* spp., *Leptospirillum* spp. and *Sulfobacillus* spp.; meanwhile, in the circumneutral sample, there was a diversity of metabolisms, aerobic and anaerobic, but with chemoheterotrophic bacteria being dominant. As this was the first survey of the Domuyo microbial community, it revealed a rich and interesting environment. We analyzed the microbial structure of a collection of hot springs around the world and found that both pH and temperature correlated with the phylogenetic distance between communities. However, only the groupings of acidic and circumneutral were representative, but not very explicative. Interestingly, the source of the data was also a significant variable, suggesting the existence of a geographic influence on the microbial community or a bias due to experimental design. In summary, the communities’ profile was shaped in a much more complex way, with factors going beyond pH and temperature conditions. Furthermore, we found no correlation between these two parameters and the alpha diversity of the communities, in disagreement with the general belief. Lastly, we found several taxa that are associated strongly with certain conditions of pH and temperature and seem widespread in hot springs, some of which are still uncultured. Therefore, genomic and phenotypic characterization of these taxa would be useful in comprehending how they survive in these extreme habitats. 

## Figures and Tables

**Table 1:** Physicochemical parameters measured in situ.

ID	Sample	pH	T [°C]	Eh [mV]	Oxygen [mg/L]	Conductivity [mS]	Arsenic [mg/L]	Sulfide [mg/L]	Iron [mg/L]
AL	Agua de Limón	2.36	71.1	−67.6	0.3	4.66	0.05	0.5	76.13
B9	Baño 9	2.43	57.0	−137.1	0.3	1.93	0.40	0.4	17.59
LT	Los Tachos	7.66	75.4	147.0	8.2	3.55	0.10	0.1	0.00

**Table 2:** Analysis of the occurrence of taxa across site conditions. The square root of the indicator value (IndVal) of the most significative taxa is presented. Components of the IndVal, specificity (A) and fidelity (B), are shown as well. Condition groups are defined as acidic (A), circumneutral (N), thermophilic (T) and hyperthermophilic (H), with combinations being considered.

Group	Taxa	√(IndVal)	A	B	*p*-Value
A	*Hydrogenobaculum*	0.87	0.99	0.77	0.0002
AH	*Sulfolobaceae* Family	0.72	0.73	0.70	0.0046
AH	*Acinetobacter*	0.64	0.89	0.47	0.0032
AT	*Desulfurella*	0.72	0.86	0.60	0.0002
AT	BSLdp215	0.68	0.99	0.47	0.0004
N	Hydrothermae Phylum	0.83	0.99	0.69	0.0002
N	*Thermus*	0.76	0.79	0.72	0.0004
N	*Caldimicrobium*	0.73	0.96	0.55	0.0002
N	OPB56	0.72	0.94	0.55	0.0002
N	*Hydrogenobacter*	0.70	0.90	0.55	0.0004
H	*Anoxybacillus*	0.57	1.00	0.32	0.006

## Supplementary Materials

The following are available online at https://www.mdpi.com/2076-2607/8/6/906/s1; Figure S1: Rarefaction curves of Caviahue-Copahue and Domuyo samples, Table S1: Hot springs collection metadata, Table S2: Alpha diversity of hot springs collection.
