# Supplementary figures and images for: Meta-Analysis of Microbial Communities in Hot Springs: Recurrent Taxa and Complex Shaping Factors beyond pH and Temperature
Source: Microorganisms. 2020 Jun 16;8(6):906. doi: 10.3390/microorganisms8060906 (PMC7356817; doi:10.3390/microorganisms8060906)

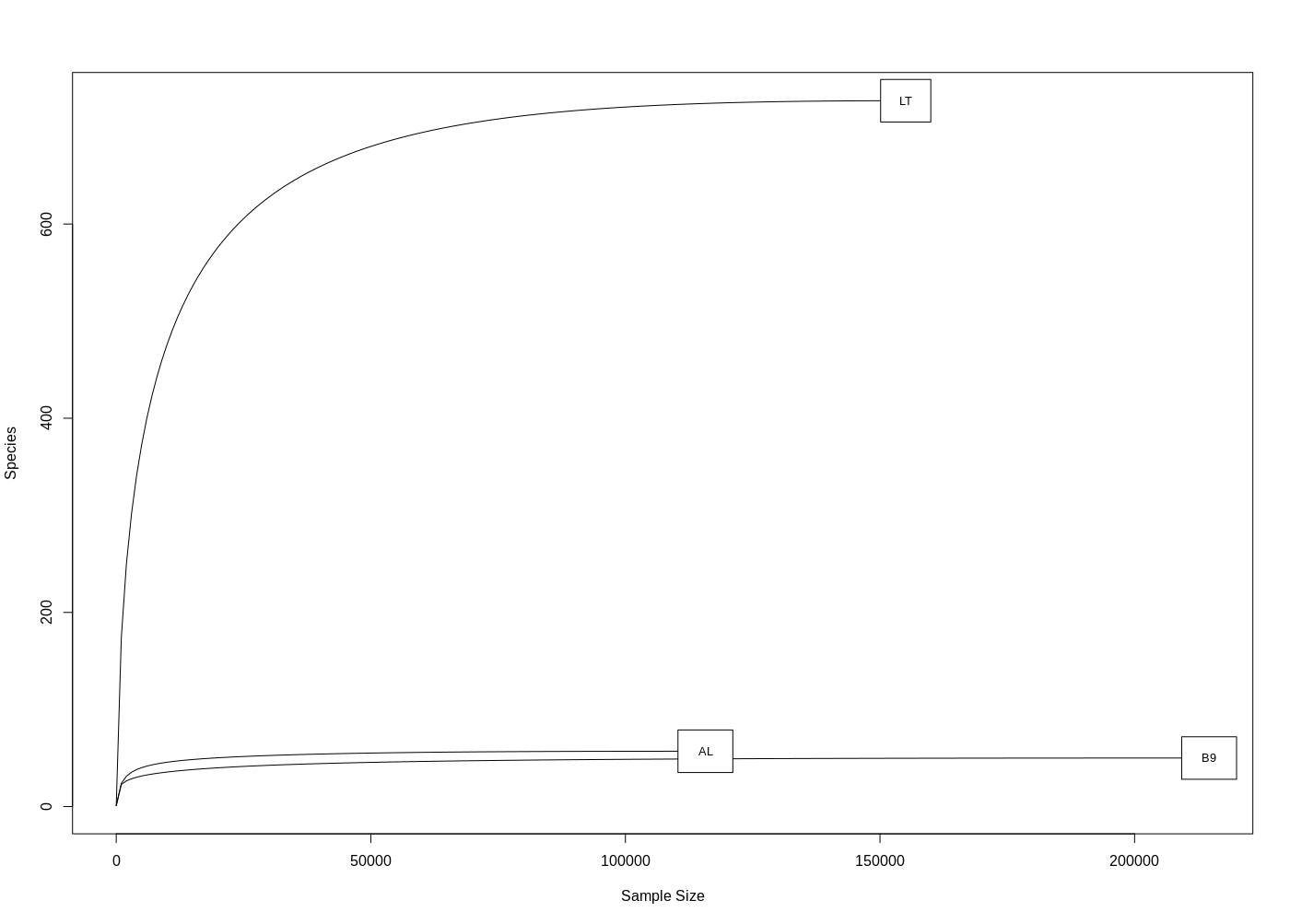

Supplement: Supplementary file 1 [file microorganisms-08-00906-s001.zip › SupplementaryFigureS1.png]
